# Small Heat Shock Protein (sHsp22.98) from *Trialeurodes vaporariorum* Plays Important Role in Apple Scar Skin Viroid Transmission

**DOI:** 10.3390/v15102069

**Published:** 2023-10-09

**Authors:** Savita Chaudhary, Vijayanandraj Selvaraj, Preshika Awasthi, Swati Bhuria, Rituraj Purohit, Surender Kumar, Vipin Hallan

**Affiliations:** 1 Plant Virology Laboratory, Division of Biotechnology, CSIR—Institute of Himalayan Bioresource Technology, Palampur 176061, Himachal Pradesh, India; 2 Academy of Scientific and Innovative Research (AcSIR), Ghaziabad 201002, Indiarituraj@ihbt.res.in (R.P.); 3 Plant Molecular Virology Laboratory, Molecular Biology and Biotechnology Division, CSIR-National Botanical Research Institute, Lucknow 226001, Uttar Pradesh, India; 4 Bioinformatics Lab, Division of Biotechnology, CSIR—Institute of Himalayan Bioresource Technology, Palampur 176061, Himachal Pradesh, India

**Keywords:** apple scar skin viroid, *Trialeurodes vaporariorum*, small heat shock proteins (sHSPs), transient silencing, tobacco rattle virus, viroid transmission

## Abstract

*Trialeurodes vaporariorum,* commonly known as the greenhouse whitefly, severely infests important crops and serves as a vector for apple scar skin viroid (ASSVd). This vector-mediated transmission may cause the spread of infection to other herbaceous crops. For effective management of ASSVd, it is important to explore the whitefly’s proteins, which interact with ASSVd RNA and are thereby involved in its transmission. In this study, it was found that a small heat shock protein (sHsp) from *T. vaporariorum*, which is expressed under stress, binds to ASSVd RNA. The *sHsp* gene is 606 bp in length and encodes for 202 amino acids, with a molecular weight of 22.98 kDa and an isoelectric point of 8.95. Intermolecular interaction was confirmed through *in silico* analysis, using electrophoretic mobility shift assays (EMSAs) and northwestern assays. The sHsp22.98 protein was found to exist in both monomeric and dimeric forms, and both forms showed strong binding to ASSVd RNA. To investigate the role of sHsp22.98 during ASSVd infection, transient silencing of *sHsp22.98* was conducted, using a tobacco rattle virus (TRV)-based virus-induced gene silencing system. The *sHsp22.98*-silenced whiteflies showed an approximate 50% decrease in ASSVd transmission. These results suggest that sHsp22.98 from *T. vaporariorum* is associated with viroid RNA and plays a significant role in transmission.

## 1. Introduction

Apple (*Malus domestica*) is a commercially important temperate fruit crop. It is commonly infected with more than 21 viruses and 8 viroids [1,2]. Apple scar skin viroid (ASSVd), which belongs to the genus *Apscaviroid* and family *Pospiviroidae*, is a major viroid infecting apple [3,4,5]. The ASSVd genome consists of circular, single-stranded RNA (ssRNA) with ~330 nucleotides, and it autonomously replicates in the host cell nucleus. ASSVd is known to cause apple scar skin and dappling diseases in apple, as well as rusty skin, fruit crinkling, and fruit dimple diseases in pear [6,7,8,9]. ASSVd spreads from infected to healthy plants naturally through grafting and mechanically through pruning tools. The viroid is known to be transmitted through seeds, but at a very low rate [10]. As the major symptoms of viroids appear on fruits, their infection decreases the quality of the fruits by producing deformation, rendering them unmarketable, which leads to direct economic loss for farmers. ASSVd disease inhibits the growth of apple saplings by altering leaf metabolism [11]. ASSVd is geographically widely distributed and has been reported in Japan, India, China, South Korea, Iran, the USA, Canada, Italy, France, Australia, and Argentina [12]. ASSVd has been reported to infect apple, wild apple, pear, peach, apricot, cherry, and Himalayan wild cherry [13,14]. ASSVd is also known to replicate in several herbaceous plants, such as cucumber, tomato, pea, eggplant, *Nicotiana benthamiana*, *N. tabacum*, *N. gluinosa*, *Chenopodium quinoa*, and *C. amaranticolar*, under experimental conditions. Infected plants show mild chlorosis and stunting symptoms [15]. Recently, it was reported that the transmission of ASSVd from infected apple trees to tree-associated fungi also occurs under natural conditions [16].

ASSVd is transmitted by *Trialeurodes vaporariorum*, commonly known as the greenhouse whitefly (WF), in herbaceous plants [17]. *T. vaporariorum* is a phloem-sap-sucking polyphagous pest that can feed on plants belonging to 82 families [18]. About 63 species of greenhouse whitefly are assigned to the genus *Trialeurodes* in the family *Aleyrodidae*. They cause damage by feeding on plants and by transmitting different viruses and viroids. *T. vaporariorum* transmits important criniviruses, i.e., potato yellow vein virus, tomato infectious chlorosis virus, tomato chlorosis virus, cucumber chlorotic spot virus, cucumber yellows virus, melon yellows virus, blackberry yellow vein associated virus, and diodia vein chlorosis virus, causing serious damage to economically important crops [19,20,21,22,23]. Apart from criniviruses, they also transmit beet pseudo-yellows virus (*Closterovirus*) and tomato necrotic dwarf virus (*Torradovirus*) [24].

Cucumber phloem protein 2 (CsPP2) and tomato viroid RNA-binding protein 1 (Virp1) have been found to interact with potato spindle tuber viroid (PSTVd) and hop stunt viroid (HSVd), respectively [25,26]. CsPP2 was also found to interact with ASSVd RNA, and its addition to the artificial diet enhances the uptake and transmission of viroid RNA by the greenhouse whitefly [17]. For replication, PSTVd binds to ribosomal protein L5 (RPL5), regulates the splicing of transcription factor IIIA (TFIIIA), and produces a splice variant named TFIIIA-7ZF, which, in turn, directs the host polymerase II (Pol II) for PSTVd replication [27,28,29]. The TFIIIA-7ZF also binds to HSVd, citrus bark cracking viroid, and apple fruit crinkle viroid, increasing their replication [30,31]. PSTVd and other members of the *Pospiviroidae* family reprogram host DNA ligase 1 to function as an RNA ligase, which helps in the circularization of viroid monomeric RNA forms [32]. HSVd binds to histone deacetylase 6 (HDA6) and reprograms it to promote epigenetic changes that lead to the transcriptional modifications observed during viroid pathogenesis [33,34]. The chloroplastic replicating *Avsunviroidae* family members utilize nuclear-encoded polymerase (NEP) for replication [35]. Limited information is available regarding the interaction between viroids and insect proteins and their role in transmission. PSTVd is known to be transmitted by aphids, i.e., *Myzus persicae*; however, transmission occurs only when plants are coinfected with potato leafroll virus (PLRV). In this case, PSTVd RNA is encapsulated by the PLRV coat protein, which leads to its uptake and transmission by aphids [36].

In a previous study, the analysis of MALDI-TOF data on ASSVd-interacting *T. vaporariorum* proteins revealed that the small heat shock protein (sHsp) interacts with ASSVd RNA [37]. Small heat shock proteins (sHsps) are molecular chaperones that contain alpha-crystallin domain (ACD) and are produced under stress conditions. The molecular weights of these proteins range from 12 to 43 kDa and lack ATPase domain. In conjunction with other heat shock proteins (Hsps), they respond quickly under stress and associate with other proteins to prevent their misfolding, aggregation, and refolding. sHsps are also known to protect messenger RNAs (mRNAs) during stress-induced translational arrest, although it is unclear whether RNA interactions with sHsps occur directly or via intermediary proteins [38,39]. sHsp15 from *Escherichia coli* is known for its binding to RNA and DNA. Its structure reveals an RNA-binding domain known as the αL motif, and it is structurally homologous to the ribosomal protein S4 and tRNA synthetase [40,41,42].

Virus-induced gene silencing (VIGS) has been widely used for functional studies of interaction, allowing for the transient silencing of target genes. The delivery of dsRNA and siRNA into insects for target gene silencing has been carried out using methods such as injection, artificial feeding, and plant delivery. Tomato plants transiently expressing truncated genes of *Bemisia tabaci*, *Cyclophilin B* (*CypB*), and *Heat shock protein 70* (*Hsp70*) through a tobacco rattle virus (TRV)-mediated VIGS system have been found to reduce tomato yellow leaf curl virus (TYLCV) transmission. However, the silencing of these genes had a deleterious effect on whitefly fitness [43]. ASSVd is acquired and transmitted by *T. vaporariorum* as a ribonucleoprotein complex [17]. However, the complex association of the viroid RNA with *T. vaporariorum* proteins, and its role in transmission, is unknown. The dynamic nature of sHsps in nucleotide binding and chaperone function prompted this study of its role in the transmission of ASSVd. In the present study, it is reported that *T. vaporariorum* sHsp22.98 interacts with ASSVd RNA, and its silencing reduces ASSVd transmission.

## 2. Materials and Methods

### 2.1. Maintenance of Whitefly Colony and Pathogen

A pure colony of *T. vaporariorum* was raised from a single fertilized female on cucumber plants. The *mitochondrial cytochrome oxidase I* (*mtCOI*) gene sequence was used to confirm whitefly identification [44]. Whiteflies were routinely checked for potential ASSVd and other viral infections in order to ensure a pathogen-free colony since our lab also conducts research on cucumber mosaic virus (CMV) and tomato leaf curl Palampur (ToLCPalV) virus. The plants and greenhouse whitefly colonies were maintained in insect-proof cages, and the glasshouse was under controlled conditions at 22 ± 1 °C, 60% relative humidity, and a 12h light/12h dark photoperiod.

### 2.2. Characterization of sHsp Gene

Based on MALDI-TOF hits, primers for amplification of the complete sHsp gene were designed. Total RNA of *T. vaporariorum* was extracted using TriZol reagent (Invitrogen, Carlsbad, CA, USA), and the first strand cDNA was synthesized using Verso cDNA synthesis kit (Thermo Fisher Scientific, Waltham, MA, USA) following the manufacturer’s instructions. The *sHsp*-gene-specific primers VH23F/VH23R were used to amplify the complete gene (Table 1). For amplification, proofreading TaKaRa LA Taq^®^ DNA polymerase (TakaraBio, Otsu, Shiga, Japan) was used. The reaction was set up as per manufacturer instructions, and for amplification, the reaction conditions used were as follows: initial denaturation for 1 min at 95 °C; 30 cycles at 98 °C/10 s; annealing at 55 °C/30 s; elongation at 68 °C/1 min.; and final elongation at 72 °C/10 min. The amplified gene was cloned into pGME^®^-T easy vector (Promega, Madison, WI, USA) and transformed into chemically competent *E. coli* cells. Positive colonies were identified using colony PCR and a combination of vector (M13 forward) and gene-specific reverse primer (Table 1). SapphireAmp Fast PCR master mix (TakaraBio) was used for amplification (initial denaturation for 1 min at 95 °C; 30 cycles consisting of 98 °C/10 s, 55 °C/20 s; and elongation at 68 °C/15 s). Two individual positive recombinant colonies were sequenced using M13 forward and reverse primers in Sanger sequencing. Details of primers used are given in Table 1.

### 2.3. Heterologous Expression and Purification of Small Heat Shock Protein

For heterologous expression, the complete *sHsp* gene was amplified by using VH42F/VH42R primers. For amplification proofreading, enzyme TaKaRa LA Taq^®^ DNA polymerase was used. The reaction conditions used are detailed in Section 2.2. The amplified product was cloned in pGEM^®^-T easy vector (Promega) and mobilized to pET28a (+) vector (Novagen, Darmstadt, Germany) using *BamH*I and *Xho*I sites (introduced through primers in gene for cloning). The recombinant pET28a-sHsp plasmid was transformed into *E. coli* BL21(DE3) strain and protein expression was induced with 0.3 mM IPTG at 37 °C for 4 h. After sonication, the insoluble pellet containing the expressed protein was dissolved in binding buffer (0.5 M NaCl, 5 mM imidazole, 20 mM Tris HCl, pH 7.9, 6 M urea). The protein was purified using Ni-NTA column chromatography (Novagen). The purified protein was dialyzed for removal of urea and run on 12% SDS-PAGE gel. Details of primers used are given in Table 1.

### 2.4. Western Blotting

Heterologously expressed purified sHsp protein was fractioned on SDS-PAGE (12%) gel, and proteins were transferred to polyvinylidene diflouride (PVDF) membrane. The membrane was incubated for 1 h in 3% blocking solution (3% *w*/*v* skim milk in PBST: 137 mM NaCl, 2.7 mM KCl, 8 mM Na_2_HPO_4_, and 2 mM KH_2_PO4 with 0.5% Tween 20) at room temperature (RT). The protein-bound membrane was further incubated for one hour with polyclonal antiserum raised in rabbit against sHsp (diluted 1:5000 in PBST) at RT. The membrane was washed with PBST three times at RT/5min and then incubated with horseradish-peroxidase-conjugated anti-rabbit immunoglobulin G antibody (1:10,000; Sigma-Aldrich, St. Louis, MO, USA) at RT/1 h. For signal development, the membrane was incubated with HRP substrate (Immobilon^®^ Forte) in the dark for 1 min/RT, and signals were captured using a chemiluminescent detector (Azure Biosystems C300, Dublin, CA, USA).

### 2.5. In Vitro Binding Assay

The in vitro binding of heterologously expressed sHsp protein to ASSVd transcript RNA was carried out using an electrophoretic mobility shift assay (EMSA) and northwestern blot. A MEGAscript^®^ T7 kit (Thermo Fisher Scientific) was used for synthesis of ASSVd transcript using *Spe*I linearized recombinant ASSVd plasmid DNA as a template [15]. The EMSA experiment was carried out by incubating different concentrations of sHsp proteins (50 to 800 ng) with 1 µg of ASSVd transcript. The RNA-protein complex was incubated at room temperature for 30 min in binding buffer (150 mM KCl, 0.1 mM dithiothreitol, 1 mM EDTA, 10 mM Tris, pH 7.4). The purified sHsp protein and ASSVd transcript were used as controls. RNA-protein complexes were separated using agarose gel electrophoresis in 2.5% agarose gel at 250 V in 0.5X TB buffer. The complex was detected by staining with ethidium bromide 0.2 µg/mL [45].

Northwestern assay was performed according to Marcos (1999), with minor modifications [46]. Briefly, the purified protein was separated on 12% SDS-PAGE and electroblotted to nitrocellulose membrane. The protein-bound membrane was incubated for 2 h at 4 °C in RN buffer (10 mM Tris–HCl, pH 7.5, 1 mM EDTA, 100 mM NaCl, 0.05% triton X-100, 1X Denhardt’s reagent), followed by 3 h incubation with digoxigenin-labeled ASSVd probe. The membrane was then washed twice with 2X wash buffer (2X SSC and 0.1% SDS) at RT for 5 min and once with 1X wash buffer (0.1X SSC, 0.1% SDS) for 15 min./65 °C. The membrane was further incubated in blocking solution (1 g blocking powder in 100 mL of 1X maleic acid buffer: 0.1 M maleic acid, 0.15 M NaCl) at 37 °C /1 h and followed by incubation with anti-digoxigenin-AP Fab fragment antibody (500 mU/mL) at 37 °C/1 h. The ASSVd interacting protein band was visualized by using one-step NBT/BCIP substrate.

### 2.6. Bioinformatic Analysis of ASSVd Association with Small Heat Shock Protein

The three-dimensional structural and dynamic association of ASSVd with *T. vaporariorum* small heat shock proteins was carried out using HADDOCK program and BIOVIA discovery studio software package. The ASSVd 330 base nucleotides (accession no. FM208138) and sHsp were used for modeling. The ASSVd viroid RNA and sHsp protein were modeled using Discovery Studio software package and then used for interaction analysis. The RNA-binding site on protein and protein-binding site on RNA were evaluated using the same package. This interaction was carried out by adopting HADDOCK program, which is governed by ambiguous interaction restraints (AIRs).

### 2.7. Transient Silencing of Small Heat Shock Protein in T. vaporariorum

Tobacco rattle virus (TRV)-based virus-induced gene silencing (VIGS) vectors (pTRV1 and pTRV2) were used for the generation of double-stranded RNA (dsRNA)/siRNA in tomato plants. The truncated *sHsp* gene (253 bp) was amplified by using VH65F/ VH65R primer pair (PCR conditions, as detailed in Section 2.2) and cloned in pTRV2 vector at *Sac*I and *Xho*I sites, named TRV2-TvsHsp. Briefly, the TRV vector containing the truncated TvsHsp22.98 region was designed to target the N-terminal region of the gene. Similarly, the truncated tomato *PDS* (*Topds*) gene (409 bp) was amplified by using primer pair To-PDS-F/To-PDS-R, cloned in TRV2 vector at *Xba*I and *BamH*I sites, and named TRV2-Topds. The TRV1, TRV2-TvsHsp, and TRV2-Topds constructs were transformed individually into *Agrobacterium tumefaciens* GV3101 strain. The bacterial suspensions were mixed at a 1:1 ratio of TRV2-TvsHsp, TRV2-Topds, and TRV2 with TRV1 and agroinfiltrated in tomato cotyledons. The TRV1:TRV2-Topds-inoculated plants were used as a visual marker for silencing, and TRV1:TRV2-mock-inoculated plants were used as mock controls. The agroinoculated plants were maintained in insect-proof cages under controlled conditions. The transient expression of TRV1 and TRV2 constructs were analyzed by employing RT-PCR using vector (TRV1- VH31F/VH31R; TRV2- VH30F/VH30R) and target-gene-specific primers. Details of primers are given in Table 1.

The non-viruliferous *T. vaporariorum* maintained on cucumber plants were starved for 2 h and then allowed to feed on tomato plants expressing TRV2-TvsHsp after 10 days post-agroinfiltration (dpa). Silencing of *sHsp* gene in whiteflies fed TRV2-TvsHsp plants was analyzed after 7 and 10 dpa. To confirm silencing, full-length primers of *sHsp* gene were used, along with *cox* gene as the internal control. Amplicons were quantified using Uvitech imaging software. Whiteflies were closely monitored for mortality, nymphal development, abnormalities, changes in behavior, growth, and development compared to non-silenced whiteflies maintained on healthy plants for three weeks. The primers used to quantify *sHsp* (VH42F/VH42R) and *cox* gene (VH25F/VH25R) are listed in Table 1. Experiments were repeated three times.

### 2.8. Transmission of ASSVd by T. vaporariorum

The *sHsp*-silenced whiteflies along with healthy controls were starved for 2 h, followed by artificial feeding with ASSVd transcript (5 µg) in 50 mL falcon tubes for viroid acquisition (6 h). The artificial diet contained 5 µg ASSVd transcript, 20% sugar solution, and 10 µg purified CsPP2. These whiteflies (viruliferous and non-viruliferous) were released on healthy cucumber plants at two leaf stages for 24 h. Later, the plants were sprayed with insecticide to kill whiteflies and kept in insect-proof cages. Whiteflies fed with 20% sugar solution and 10 µg purified CsPP2 were used as healthy controls. The whiteflies were later released on healthy cucumber plants and tested for transmission of ASSVd at 14 dpi using RT-PCR with viroid-specific primers (VH40F/VH40R) (Table 1). Statistical analysis was conducted using an unpaired t-test to assess the significance of the transmission efficiency of healthy WFs and silenced WFs. Experiments were repeated three times.

## 3. Results

### 3.1. Molecular Characterization of T. vaporariorum Small Heat Shock Protein (sHsp22.98)

The full-length *T. vaporariorum sHsp* gene that was amplified from whitefly cDNA resulted in a specific ~600 base pair (bp) amplicon. The sequence analysis showed the *sHsp* gene is 606 bp in length and encodes for 202 amino acids, with a molecular weight of 22.98 kDa. ProtParam (incorporated in the Expasy translate tool) predicted the isoelectric point of sHsp is 8.95. The molecular weight of the identified sHsp protein was 22.98, and based on this, it was named sHsp22.98. Blastn and Blastx analysis showed the highest homology at 96.67% and 99.41% with the *T. vaporariorum* small heat shock protein (FJ183792.1), respectively. sHsp was found to contain the conserved alpha-crystallin domain (77–153 aa), identified using a conserved domain database search in NCBI. The sHsp22.98 protein contains only one cysteine residue (42 aa) and is present toward the N-terminal of the alpha-crystallin domain. This residue is considered important for the dimerization of protein. The sequence was submitted to the NCBI database under the following accession number: MT557571.1.

### 3.2. Recombinant Expression of sHsp22.98

**Figure 1:**
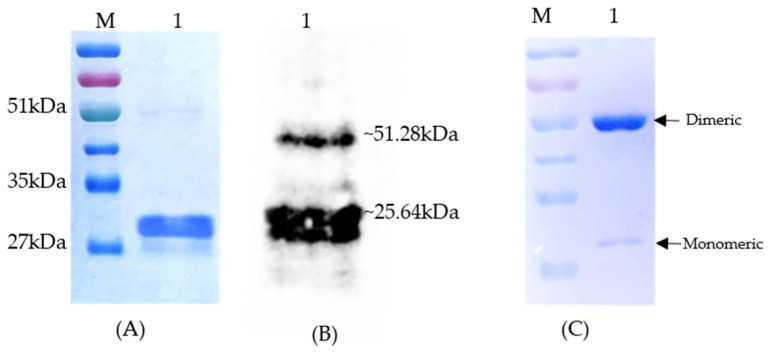
Heterologous expression and purification of sHsp22.98 from *T. vaporariorum*. (**A**) Expression and purification of sHsp22.98 in *E. coli* BL21 (DE3) cells (lane 1). (**B**) Detection of monomeric and dimeric forms of purified sHsp22.98 by Western blotting using sHsp22.98-specific antisera (lane 1). (**C**) Dimerization of recombinant sHsp22.98 after storage. Lane 1: purified sHsp22.98 protein fractionated on SDS-PAGE. Lane M: protein ladder (3-color prestained protein ladder, 10–250 kDa, Genetix).

The 606 bp *sHsp22.98* gene was cloned in a pET28a (+) vector and in-frame insertion was confirmed by sequencing. Sequence analysis of the pET28a-sHsp22.98 recombinant plasmid showed that the sHsp22.98 gene was in frame with the His-tag. The *E. coli* BL21 (DE3) cells containing the sHsp22.98-pET28a (+) plasmid exhibited the expression of a ~25.6 kDa protein after induction with 0.3 mM IPTG at 37 °C, whereas no expression was observed in uninduced cells. The expressed recombinant protein was purified by using a Ni-NTA His-binding resin column (Figure 1A). The purified protein was used as an antigen for antisera production in rabbits. The purified protein was checked with sHsp22.98 specific antibody, which showed signals specific to both sHsp22.98 monomeric and dimeric forms (Figure 1B). The dimeric form was more pronounced after storage of protein over time (~1 week at −20 °C) when separated on 12% SDS-PAGE (Figure 1C).

### 3.3. In Vitro Binding of Recombinant sHsp22.98 to ASSVd RNA

**Figure 2:**
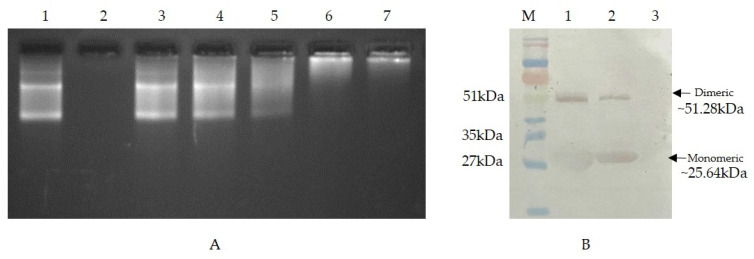
In vitro interaction of sHsp22.98 with ASSVd RNA using electrophoretic mobility shift assay (EMSA) and northwestern blot. (**A**) EMSA: RNA-protein complex was fractionated on 2.5% agarose gel. Lane 1: 1 µg ASSVd RNA; lane 2: 800 ng protein; lane 3: ASSVd RNA+ 50 ng protein; lane 4: ASSVd RNA + 100 ng protein; lane 5: ASSVd RNA + 200 ng protein; lane 6: ASSVd RNA + 400 ng protein; lane 7: ASSVd RNA + 800 ng protein. (**B**) In vitro interaction analysis of sHsp22.98 protein with digoxigenin-labeled ASSVd probe using northwestern blotting. Lane M: prestained protein ladder (Genetix). Lane 1: 10 µg of purified protein, lane 2: 5 µg of purified protein, lane 3: BSA as negative control.

The binding properties of sHsp22.98 protein to the ASSVd transcript were confirmed by in vitro RNA-protein interaction detection assays *viz.*, EMSA and northwestern blot. In EMSA, the migration of the sHsp22.98-ASSVd RNA-bound complex was checked with varying concentrations of sHsp22.98 protein (50 ng to 800 ng: lane 3–lane 7) (Figure 2A). At all tested concentrations, the migration of the sHsp22.98-ASSVd RNA complex was retarded in agarose gel electrophoresis. Significant retardation in migration was observed at 400 ng and 800 ng of protein (lanes 6 and 7; Figure 2A). In northwestern assays, heterologously expressed recombinant sHsp22.98 protein showed binding to digoxigenin-labeled ASSVd RNA, and the interaction was found to both monomeric and dimeric forms (Figure 2B). The results obtained in EMSA and northwestern assays confirm the binding properties of sHsp22.98 to ASSVd RNA. 

### 3.4. In Silico Analysis of sHsp22.98 Dynamic Association with ASSVd

**Figure 3:**
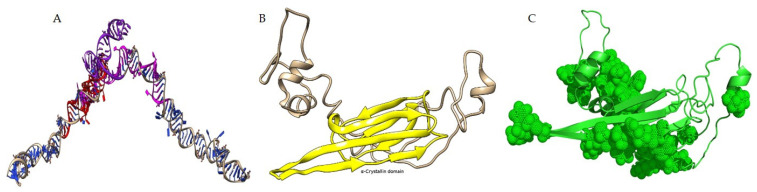
Molecular modeling and prediction of binding sites in ASSVd and *T. vaporariorum* small heat shock protein22.98 (sHsp22.98). (**A**) Three-dimensional structure of ASSVd RNA showing three protein-binding sites. Purple: BS1; magenta: BS2; red: BS3. (**B**) Structure of sHsp22.98 monomer showing the presence of alpha-crystalline domain in yellow. (**C**) Three-dimensional structure of sHsp22.98 monomer protein amino acids showing predicted RNA-binding sites in circular dots. The ASSVd RNA and sHsp protein were modeled using Discovery Studio software package.

**Figure 4:**
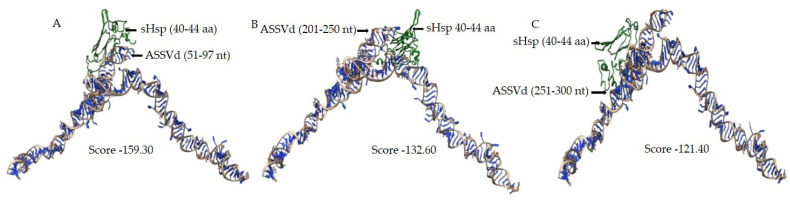
Dynamic association of sHsp22.98 predicted protein-binding sites to ASSVd**.** The predicted interacting position of ASSVd and sHsp22.98 are given in brackets. The free energy of the interaction between ASSVd and sHsp22.98 is listed as a score. The ASSVd–sHsp22.98 interaction was carried out by adopting RNA homology modeling protocol built in Discovery Studio software package. (**A**) sHsp22.98–site3 and RNA–BS1 complex (51–97), with a score of −159.30. (**B**) sHsp22.98–site3 and RNA–BS2 complex (201–250), with a score of −132.60. (**C**) sHsp22.98–site3 and RNA–BS3 complex (251–300), with a score of −121.40.

**Figure 5:**
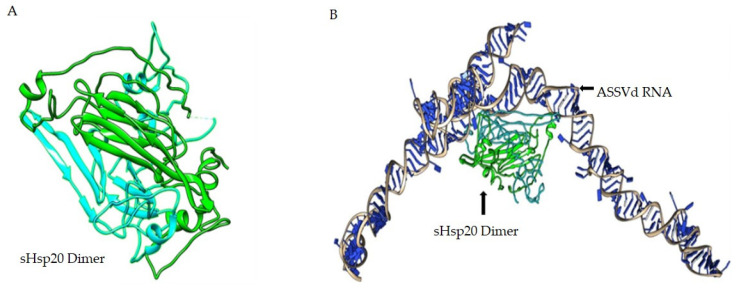
Dynamic association of sHsp22.98 dimer with ASSVd RNA. (**A**) The predicted dimeric form of sHsp22.98. (**B**) The predicted interacting position of ASSVd and dimeric form of sHsp22.98. The ASSVd-sHsp22.98 interaction was carried out by adopting RNA homology modeling protocol built in Discovery Studio software package.

The ASSVd and sHsp22.98 (MT557571) three-dimensional structures were obtained using the BIOVIA Discovery Studio software package (Figure 3). The modeled structures of ASSVd and sHsp22.98 were used to establish the dynamic interaction and identification of binding sites. The interactions were carried out using the HADDOCK program. Three protein-binding sites (BSs) in ASSVd RNA—BS1 at nucleotide position 51–97 (purple colored), BS2 at nucleotide position 201–250 (magenta), and BS3 at nucleotide position 251–300 (red)—were identified (Figure 3A). The alpha-crystallin domain (77–153 aa, yellow-colored) lies in the center (Figure 3B). In the case of the sHsp22.98 protein, fifteen RNA-binding sites at amino acid positions 3–6, 27, 40–44, 47, 56–59, 61–62, 69–72, 75–80, 116–118, 122–128, 154, 156–157, 159, 178–181, and 189–196 were identified (Figure 3C). The sHsp22.98-site3 (40–44 aa) showed more affinity for ASSVd RNA. The sHsp22.98-site3 was further analyzed with the three available ASSVd RNA-binding sites. The sHsp22.98-site3 and RNA-BS1 (51–97) complex had the highest free energy, with a score of −159.30, showing the highest affinity for viroid RNA (Figure 4A). RNA-BS2 and BS3 also showed interactions with site3, but their free energy was less, i.e., −132.60 and −121.40, respectively (Figure 4B,C). sHsps form large heterooligomeric aggregates and are known to form dimers. The dimeric structure of sHsp22.98 was also predicted, and the dimeric form of sHsp22.98 showed affinity (−31.25) for ASSVd (Figure 5A,B). These results complement the EMSA and northwestern assays, where an ASSVd RNA interaction was observed with monomeric and dimeric forms of the sHsp22.98 protein.

### 3.5. Plant-Mediated Silencing of sHsp22.98 in T. vaporariorum

**Figure 6:**
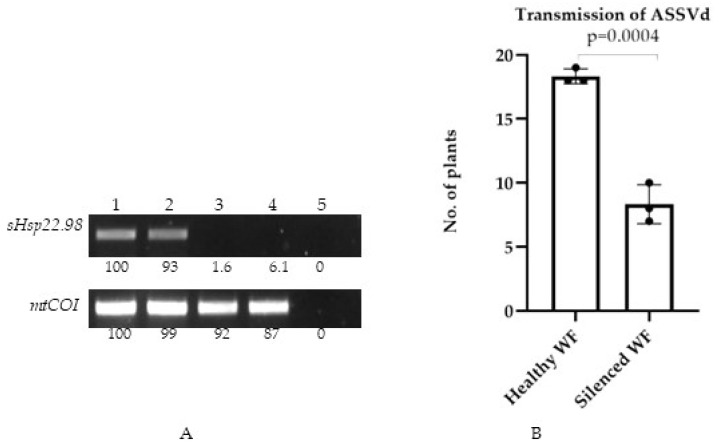
Quantification of *sHsp22.98* mRNA and effect of ASSVd transmission by sHsp22.98-silenced whiteflies. (**A**) Quantification of target *sHsp22.98* mRNA using semiquantitative PCR from control (lane 1 and lane 2) and *sHsp22.98*-silenced whiteflies (lanes 3 and 4); lane 5: non-template control. *mtCoI* gene was used as internal control. Amplicon intensity was quantified using Uvitech software, and healthy whiteflies (lane 1) were used as the control for quantification. Values represent the percent expression. (**B**) Transmission of ASSVd by *sHsp22.98*-silenced whiteflies.

The *mtCOI* gene was used to determine the identity of the greenhouse whiteflies. In sequencing, the amplicon shows 100% identity with the *T. vaporarium* gene, confirming the identity of the colony. The healthy greenhouse whitefly colonies were also checked for cross-contamination of viroid, CMV, and ToLCPalV, and the colony was found to be free from these contaminants. TRV-mediated transient expression of Tv-sHsp22.98 in tomato was confirmed using RT-PCR at 10 and 14 dpi. The amplification of the desired bands from the upper uninoculated leaves confirmed the systemic movement and replication of the TRV-TvsHsp22.98 in tomato plants. The tomato *PDS* gene (*phytoene desaturase*) was used as a visual marker for the observation of silencing, as silencing of this gene leads to photobleaching in leaves (Figure S1). The targeted dsRNA forms were delivered to healthy whiteflies by feeding them TRV-TvsHsp22.98 plants after 14 days post-agroinfiltration, aiming to achieve silencing of the target gene (sHsp22.98). Whiteflies were allowed to feed on these plants for 10 days. The whiteflies were checked at 7 and 10 dpi for silencing of the *sHsp22.98* gene. On day 10, no amplification for the *sHsp22.98* gene was observed (lanes 3 and 4, Figure 6A). Plant-mediated silencing led to a reduction in the expression of the *sHsp22.98* transcript by ~94 to 98%. The cox gene was used as an internal control for expression comparison. The *sHsp22.98*-silenced whitefly did not show any significant morphological changes based on visual and microscopic observations. The reproduction of silenced whiteflies was analogous to control whiteflies. These results showed that, at 10 dpi, effective silencing of the target gene in *T. vaporariorum* was observed and silencing of *sHsp22.98* had no deleterious effect on whiteflies.

### 3.6. Transmission of ASSVd through sHsp22.98-Silenced Whiteflies

The *sHsp22.98*-silenced whiteflies and non-silenced whiteflies (used as the positive control) were artificially fed with dimeric ASSVd RNA transcripts for 6 h and released on healthy cucumber plants to study the role of sHsp22.98 in viroid transmission. Both silenced and non-silenced whiteflies were allowed to feed on plants for 24 h (20 plants each), and 7–10 whiteflies were released per plant. After 24 h, whiteflies were killed via the spraying of insecticide. Out of the 20 plants, 7 plants were found positive for ASSVd infection when the viroid was transmitted through sHsp22.98-silenced whiteflies, whereas 19 plants were found positive in the control whiteflies. The experiment was repeated three times. Overall, in this study, we found that silencing of sHsp22.98 significantly reduces the ASSVd transmission rate by up to 50% (Figure 6B). These results show that sHsp22.98 of *T. vaporariorum* plays an important role in the transmission of ASSVd.

## 4. Discussion

Viroids are the smallest known infectious non-coding, circular, single-stranded RNA molecules (234–401 nt) that infect many plant hosts, replicate autonomously, and cause important diseases [47,48,49]. Viroid RNAs display extensive internal base pairing, which leads to the formation of metastable rod-like or quasi-rod-like conformation [50]. Long-distance movement of viral and viroid RNA through phloem cells is well known for the establishment of systemic infection. CsPP2 binds to HSVd RNA as a ribonucleoprotein (RNP) complex, and this RNP complex can move from infected rootstock to non-infected scions [26,51]. These features and others may indicate that viroid RNA interacts with host factors for the development of disease. Viroids are mechanically transmissible through seeds, pollen, and insect vectors. Insect vectors, specifically whiteflies and aphids, feed on phloem, take up the virus/viroid, and transmit it to healthy plants. For example, *Dysaphis plantaginea* transmit apple chlorotic fruit spot viroid (ACFSVd); *Myzus persicae* transmits ACFSVd, PSTVd, peach latent mosaic viroid (PLMVd), and tomato planta macho viroid (TPMVd); and *Macrosiphum euphorbiae* transmits PSTVd [36,52]. The molecular mechanisms enabling viroid transmission via insect vectors have not been fully investigated, especially the identification of vector components interacting with the viroid RNA. The identification of viroid-interacting vector proteins helps to elucidate the mechanism of viroid replication, movement, and transmission.

*T. vaporariorum* is also known to transmit ASSVd in herbaceous plants, and transmission increases after feeding on *Cucumis sativus* phloem exudates and purified recombinant CsPP2 proteins [15,17]. Vector-mediated transmission may lead to the spread of ASSVd in different crops, especially in sub-temperate regions.

Finding insect proteins that interact with ASSVd RNA was the goal of this investigation. The sHsp22.98 protein of *T. vaporariorum* was found to interact with ASSVd in the present study. This interaction was confirmed using in silico analysis, EMSA, and northwestern assays. ASSVd RNA contains five domains (terminal left: TL, pathogenicity related: P, central: C, variable: V, and terminal right: TR) and two conserved motifs in the C region and the terminal conserved region (TCR) [53]. Bioinformatic analysis predicted three protein-binding regions, named binding site 1 (BS1) (51–97), BS2 (201–250), and BS3 (251–300) in the ASSVd genome. The predicted sHsp22.98 binding sites are partially complementary in the secondary rod-shaped structure of viroid RNA. The BS2 region 215–250 nt and BS3 251–273 nt are complementary to BS1 region, i.e., 51–97. The BS1 region showed the strongest binding to sHsp22.98, and this region is rich in AU sequences. sHsps is known to bind to AU-rich RNA elements (AREs), which are responsible for mRNA stability. Mammalian sHsp70 and sHsp110 are known to bind to AREs and regulate their folding, translation, and degradation [54]. Moreover, Hsp70 binds to its own mRNA, interferes with the ubiquitination pathway of mRNA decay, and regulates its expression and translation during heat shock [55,56,57,58]. Apart from this, Hsp60, 40, and 42 and sHsps10, 12, and 26 of *Saccharomyces cerevisiae*; sHsps12.2 and 16.11 as well as sip-1 of *Caenorhabditis elegans*; and sHsps26 and 27 of *Drosophila melanogaster* are known to bind to RNA [53]. Moreover, sHsp with an isoelectric point of more than 8 is known to bind to nucleotides [59]. The sHsp22.98 identified in this study also has an isoelectric point of 8.9; based on this, it can be assumed as another feature for RNA-binding. These studies showed that sHsps are important RNA-binding proteins and regulate their stability, expression, and translation.

sHsp22.98 was found to exist in both monomer and dimer forms, with both interacting with ASSVd RNA. sHspB1 is known to form monomer and dimer forms and is present in the cytosol and mitochondrial membrane of HeLa cells. Although the ratio of monomer/dimer forms at cystol is almost equal, in the mitochondrial membrane, it is up to five-fold higher [60]. Also, sHsps are well known to form hetero-oligomers. Based on this evidence, it seems that both dimeric and hetero-oligomer forms are important for their functioning. Apart from this, CsPP2 was shown to interact with ASSVd and HSVd RNA, and it also existed in monomer and dimer form [17,26,51]. VirP1, also known as bromodomain-containing host protein (BRP1), of tomato, binds to PSTVd at the TR domain. The VirP1 protein contains a nuclear localization signal (NLS) and bromodomain and is predicted to be involved in the transportation of PSTVd to the nucleus [25]. Later, it was found that VirP1 also binds to both PSTVd C-loop and importin alpha protein for its transportation to the nucleus [61]. VirP1 has also been reported in the transportation of citrus exocortis viroid (CEVd) to the nucleus [25,62]. BRPI/VirP1 was also found to be involved in the transportation of cucumber mosaic virus Q-satRNA to the nucleus and aids in its replication [63]. Co-infection of CMV and its satellite RNA results in its resistance against PSTVd [64,65], which is probably due to the sequestration of VirP1 by both the virus and its satellite RNA. Based on these studies, it can be concluded that both monomeric and dimeric forms of proteins show RNA-binding properties. Also, the viroid RNA depends on multiple host and vector factors for the establishment and spread of infection.

A number of insect proteins are known to play a role in the transmission of viruses. For example, silencing of *cyclophilin B* and *heat shock protein 70* (*Hsp70*) of *B. tabaci* resulted in 43% and 12% reductions, respectively, in tomato yellow leaf curl virus transmission [43]. In the case of cucumber vein yellowing virus (Family *Potyviridae*), amino acids 93–105 of the CP play an important role in *B. tabaci*-mediated transmission. Deletion of this region leads to the abolishment of *B. tabaci*-mediated virus transmission [66]. In our study of the role of sHsp22.98 in viroid transmission, we found that the *sHsp22.98* gene was transiently silenced by feeding greenhouse whiteflies tomato plants inoculated with TRV-TvsHsp at 10 dpi. Silenced whiteflies showed a significant reduction (50%) in the transmission of ASSVd. These results show that sHsp22.98 plays an important role in ASSVd transmission.

In summary, this study showed that viroid RNA not only interacts with host factors but is also capable of interacting with its vector proteins. Here, it was found that sHsp22.98 associates with ASSVd RNA. sHsps is a multifunctional protein that plays an important role in biotic and abiotic stress. Transient silencing of sHsp22.98 leads to a reduction in ASSVd transmission.

## Figures and Tables

**Table 1:** Details of primers used for amplification and their target region.

Primer Name	Primer Sequence	Target Region	References
VH23F	ATGGCATCTTTGAGAAGTCTCT	For sHSP22.98 amplification	In this study
VH23R	TTATGATTCCATTTTGTCACCTTTG		
M13-F	GTAAAACGACGGCCAGT	For sequencing	In this study
M13-R	CAGGAAACAGCTATGAC		
VH25 F	GGACACTCGCGCTTACTTTAC	For mtCOI amplification	[44]
VH 25 R	CAACATAACGTCGAGGCATCC		
VH40 F	GTCGACGACGACAGGTGAGTT	For ASSVd amplification	[17]
VH40 R	GTCGACGAAGGCCGGTGAGAA		
VH42 F	**GGATCC**ATGGCATCTTTGAGAAGTCTCT	For sHsp22.98-pET28a construct	In this study
VH42 R	**CTCGAG**TTATGATTTCATTTTGTCACCTTTGC		
VH65F	**GAGCTC**CAGCACTTTGGAATCGGTTTG	For tvHsp22.98-TRV construct	In this study
VH65R	**CTCGAG**GTTTGGCTTCGACGACTAGAT		
To-PDS-F	CG**GTCTAG**AGGCACTCAACTTTATAAA CC	For To-PDS-TRV construct	In this study
To-PDS-R	CG**GGGATC**CCTTCAGTTTTCTGTCAAACC		
CMV-F	ACCCTGAAACCGCCTGAAAT	For detection of CMV	In this study
CMV-R	TCCGAACTGTAACCCACACG		
ToLCPalV-F	ATGGTGAAGCGTCCAGCAG	For detection of ToLCPalV	In this study
ToLCPalV-R	TTAATTTGTTACCGAA		

Bold and underlined sequences are the restriction sites used for directional cloning.

## Data Availability

The data presented in this study are available upon request from the corresponding author.

## Supplementary Materials

The following supporting information can be downloaded at: https://www.mdpi.com/article/10.3390/v15102069/s1, Figure S1: Generation of dsRNA through VIGS-TRV system: (A) Silencing of tomato *PDS* gene (*phytoene desaturase*). (B) Mock inoculated plants (TRV-1 and TRV-2). (C) TRV-Tv sHsp22.98-infiltrated plants. The pictures shown were taken at 3 weeks post-agroinfiltration
