# Supplementary material for: Small Heat Shock Protein (sHsp22.98) from Trialeurodes vaporariorum Plays Important Role in Apple Scar Skin Viroid Transmission
Source: Viruses. 2023 Oct 9;15(10):2069. doi: 10.3390/v15102069 (PMC10611230; doi:10.3390/v15102069)
Supplement: Supplementary file 1 [file viruses-15-02069-s001.zip › viruses-2519129-supplementary.pdf]

Supplementary Material

Plant mediated silencing of sHSP in *T. vaporariorum*:

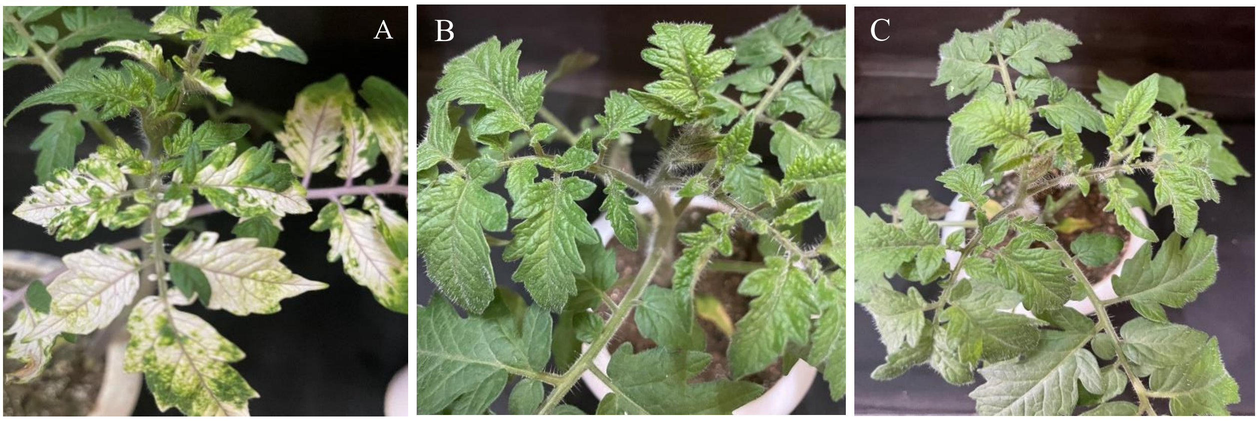

Supplementary Figure S1**.** Generation of dsRNA through VIGS-TRV system: (A) Silencing of Tomato *PDS* gene (*phytoene desaturase*) (B) Mock inoculated plants (TRV-1 and TRV-2) (C) TRV-Tv sHsp22.98 infiltrated plants. The pictures shown were takes 3 weeks post agroinfiltration.
